# Early Detection of Ultra High Risk for Psychosis in a Norwegian Catchment Area: The Two Year Follow-Up of the Prevention of Psychosis Study

**DOI:** 10.3389/fpsyt.2021.573905

**Published:** 2021-02-24

**Authors:** Inge Joa, Jone Bjornestad, Jan Olav Johannessen, Johannes Langeveld, Helen J. Stain, Melissa Weibell, Wenche ten Velden Hegelstad

**Affiliations:** ^1^TIPS—Network for Clinical Research in Psychosis, Department of Psychiatry, Stavanger University Hospital, Stavanger, Norway; ^2^Faculty of Health, Network for Medical Sciences, University of Stavanger, Stavanger, Norway; ^3^Department of Social Studies, Faculty of Social Sciences, University of Stavanger, Stavanger, Norway; ^4^Department of Psychiatry, District General Hospital of Førde, Førde, Norway; ^5^School of Arts and Humanities, Edith Cowan University, Joondalup, WA, Australia

**Keywords:** ultra high risk, at risk mental state, prodromal, psychosis, schizophrenia, early detection

## Abstract

**Objectives:** Most individuals experience a relatively long period of sub-clinical psychotic like symptoms, known as the ultra high risk (UHR) or at risk mental states (ARMS), prior to a first episode of psychosis. Approximately 95% of individuals who will later develop psychosis are not referred to specialized clinical services and assessed during the UHR phase. The study aimed to investigate whether a systematic early detection program, modeled after the successful early detection of psychosis program TIPS, would improve the detection of help-seeking UHR individuals. The secondary aim was to examine the rates and predictors of conversion to psychosis after 2 years.

**Method:** The overall study design was a prospective (2012–2018), follow- up study of individuals fulfilling UHR inclusion criteria as assessed by the structural interview for prodromal syndromes (SIPS). Help-seeking UHR individuals were recruited through systematic early detection strategies in a Norwegian catchment area and treated in the public mental health services.

**Results:** In the study period 141 UHR help-seeking individuals were identified. This averages an incidence of 7 per 100,000 people per year. The baseline assessment was completed by 99 of these and the 2 year psychosis conversion rate was 20%. A linear mixed-model regression analysis found that the significant predictors of conversion were the course of positive (0.038) and negative symptoms (0.017). Age was also a significant predictor and showed an interaction with female gender (<0.000).

**Conclusion:** We managed to detect a proportion of UHR individuals in the upper range of the expected prediction by the population statistics and further case enrichment would improve this rate. Negative symptoms were significant predictors. As a risk factor for adverse functional outcomes and social marginalization, this could offer opportunities for earlier psychosocial intervention.

## Introduction

The onset of psychosis can be devastating and typically occurs in late adolescence or early adulthood (1). This is a critical developmental stage for relationships, education and employment, and illness onset can threaten the potential for a productive, inclusive, and fulfilling adult life. Early detection programs such as the Scandinavian early treatment and intervention in psychosis (TIPS) study have endeavored to improve long term outcomes associated with psychosis by detecting and intervening early in the course of illness (2, 3). The TIPS early detection program showed significantly improved functional outcomes 10 years post diagnosis (4). The success of such programs has fueled interest in examining the feasibility of detecting psychosis at an even earlier stage, thereby further reducing disability (secondary prevention), or even averting manifestation of psychosis itself (primary prevention) (5, 6).

Prior to a first episode of psychosis (FEP), most individuals experience an extended period of sub-clinical psychotic-like symptoms, known as the ultra high risk (UHR) state (7), clinical high risk for psychosis (CHR-P) (8), or at risk mental state (ARMS) for psychosis (9). When assessed, a UHR state is usually defined as meeting criteria for one or more of three syndromes: the attenuated positive symptoms (APS) syndrome, the brief limited intermittent psychotic symptoms (BLIPS) syndrome, or the genetic risk and/or the deterioration (GRD) syndrome (10). These syndromes are assessed by validated measures, such as the Comprehensive Assessment of At-Risk Mental States (CAARMS) (11) or the Structured Interview for Prodromal Syndromes (SIPS) (7). Moreover, in the fifth version of the Diagnostic and Statistical Manual of Mental Disorders (DSM 5), APS was included as a condition for further study (12).

The UHR state is associated with high rates of experienced trauma, victimization such as bullying, substance use, comorbid mental disorders, suicidal ideation, and self-harm (1, 8, 13). While UHR individuals are more likely to be male (58%) (1), female gender is a predictor of poorer outcomes in terms of general psychiatric symptoms and functioning at long term follow up (14). Further, UHR is associated with a 22% risk of developing a psychotic disorder within 3 years (1). It should also be noted that the BLIPS syndrome overlaps diagnostically with the DSM-5 category of brief psychotic episodes (15). Up to 60% of UHR individuals who do not develop psychosis continue to display subthreshold psychotic symptoms, or meet criteria for other mental health disorders (16–18), and impairments in social functioning are as common as in other mental disorders (1).

Each of the three UHR syndromes includes declining psychosocial functioning, and often leads to the person seeking help (19). However, help seeking appears to be delayed with a recent comprehensive review of 42 meta-analyses reporting a mean duration of nearly 2 years of APS prior to accessing specialized services (1). The vast majority of UHR individuals (95%) are neither referred to UHR services nor assessed during the UHR phase (20, 21). It is likely that the overall burden of psychosis risk in secondary mental health care is mostly unknown. Clinician referrals of UHR individuals to specialized mental health services depend largely on subjective judgments of psychosis risk (22). While semi-structured interviews for psychosis prediction such as the SIPS (7) have an excellent overall prognostic performance, and the sensitivity is high (95%), the specificity is low (47%) (23). Thus, UHR criteria are still unable to correctly predict transition in all individuals who will later develop psychosis (22). There will always be challenges when attempting to detect cases of a low incidence illness stemming from populations with low or moderate risk, or, in this case, probably with no or few UHR symptoms (24, 25). This limits the predictive utility of the UHR concept outside clinical samples that have undergone risk enrichment (1).

The Prevention of Psychosis study (POP) (26) was launched in 2012 and builds on the 23 years of experience of the TIPS study (3, 27) in which intensive awareness and information campaigns were combined with low threshold detection and assessment. The awareness and information campaigns were key to the success of TIPS (2). In POP, the original psychosis specific information campaigns were adapted to encompass a broader range of symptoms and psychological distress consistent with the UHR criteria.

## Aims and Research Questions

The aim of this study was to investigate whether a systematic early detection program (POP) modeled on the successful early detection of psychosis program of TIPS (27), would improve the detection rate of persons at UHR for psychosis. We formulated the following research questions:

1. What proportion (based on other UHR studies) of the expected UHR cases can be identified through systematic early detection strategies?
2. What are the clinical characteristics for a UHR sample over a 2 year follow-up in an early detection program?
3. What are the rates and predictors of 2 year conversion to psychosis in an early detection program?

## Design

The overall study design was a prospective 2 year follow-up study of UHR individuals. UHR individuals in the catchment area of Stavanger University Hospital, Norway were recruited through intensified case detection within secondary mental health clinical services and in the general population. Consenting participants received tailored individualized treatment within the secondary mental health services and structured follow-up assessments.

## Methods

### Information Campaigns and Detection Team

The study used a health service design modeled after the TIPS study, utilizing the opportunities afforded by the Norwegian catchment area based public treatment system. In this system, specialized mental health inpatient and outpatient care serves the entire population of the catchment area. Since the mid-1990s, Stavanger University Hospital has applied intensive multi-modal and multi-level information campaigns to enhance low threshold access to services for individuals experiencing signs of FEP in order to reduce the duration of untreated psychosis (DUP) (2, 3). These have been aimed at the public as well as targeted groups such as teachers, General practitioners (GP) and primary care health personnel. The TIPS study included a low threshold detection team (DT) located in the specialized mental health care system, for assessing signs of FEP. The launch of the POP study in 2012 changed the content of these information campaigns from a focus on secondary preventions strategies (DUP reduction) to a primary prevention strategy aimed at reducing the incidence of new FEP cases (6). The broad scope of these public mental health awareness campaigns promotes mental health literacy and can therefore be considered a combination of a proximal high risk approach and a population based approach, targeting distal risk factors for adverse mental health outcomes. The DT was a vital part of the POP case detection. When the POP study started the DT combined low-threshold assessment and case identification of both potential UHR and FEP individuals.

The POP information campaigns used three specific strategies:

- Targeted information to the general public about mental health in adolescence, including the early signs of UHR, where to get help for psychological distress, the importance of getting help early, and the availability of the low threshold psychiatric DT. Information was provided through the use of regular ads in local newspapers, colorful brochures at General Practitioner (GP) practices, schools, universities, community health centers, and the like; advertisements on newspaper web-pages including a link to our website www.tips-info.com; cinema advertisements before the movie show, and Facebook advertisements. An example of the content of the information concerns people were told to be alert if a person started withdrawing from family, friends or colleagues. A 2012 quote from a newspaper advertisement aimed at teenage parents, may serve as an example: “Teenagers commonly alter behavior during puberty. However, distinct and sudden behavior changes in teenagers might reasonably worry parents. Be on guard if a teenager starts self-isolating, being uncommunicative or depressed.” All advertisements and information prominently featured contact info for our early detection team.
- Targeted education programs for teachers, GPs, and other workers in community healthcare about mental health in adolescence, UHR signs and symptoms, and the content and availability of UHR assessment and treatment. During the project period all upper secondary schools in the catchment area were annually visited by members of the DT. A 45 min lecture was offered to the teachers, focusing on the rationale, and core elements of the POP study and the availability of low-threshold UHR assessments. In addition, all GPs in the area were sent annual newsletters with greetings and thanks around Christmas and offered site visits and workshops/lectures about UHR/psychosis and drug abuse.
- Provision of targeted education for clinicians in the specialized mental health services about signs and symptoms of UHR phase. All out-patient clinics, community mental health centers, and hospital mental health wards in the catchment area were annually visited and offered education on UHR and psychosis assessment and treatment. Newly employed doctors and psychologists were offered seminars focusing on SIPS, PANSS, and SCID as part of their specialist education.

### Sample

The primary inclusion criterion was meeting one or more of the following UHR criteria: (1) lifetime Attenuated Positive Symptoms (APS); (2) lifetime Brief Limited Intermittent Psychotic Symptoms (BLIPS) or (3) lifetime Genetic Risk and Deterioration syndrome (GRD). Further inclusion criteria were: living in the catchment area; aged between 13 and 65 years; Norwegian or other Scandinavian language speaking; and ability to provide written informed consent. The exclusion criteria were: lifetime or current use of antipsychotic medication for more than 4 weeks; any known neurological or endocrine disorders; lifetime or current occurrence of psychosis and intellectual functioning below IQ of 70.

The FEP treated incidence rate in the Stavanger University hospital catchment area, measured by the TIPS studies is 17–19 per 100,000 people (2, 28, 29). Based on these figures and the 1-year psychosis conversion risk of 20% (30), we estimated the yearly incidence of UHR in our catchment area (~300,000 inhabitants fulfilling age criteria for inclusion) as 90 per 100,000 people.

### Recruitment

**Figure 1:**
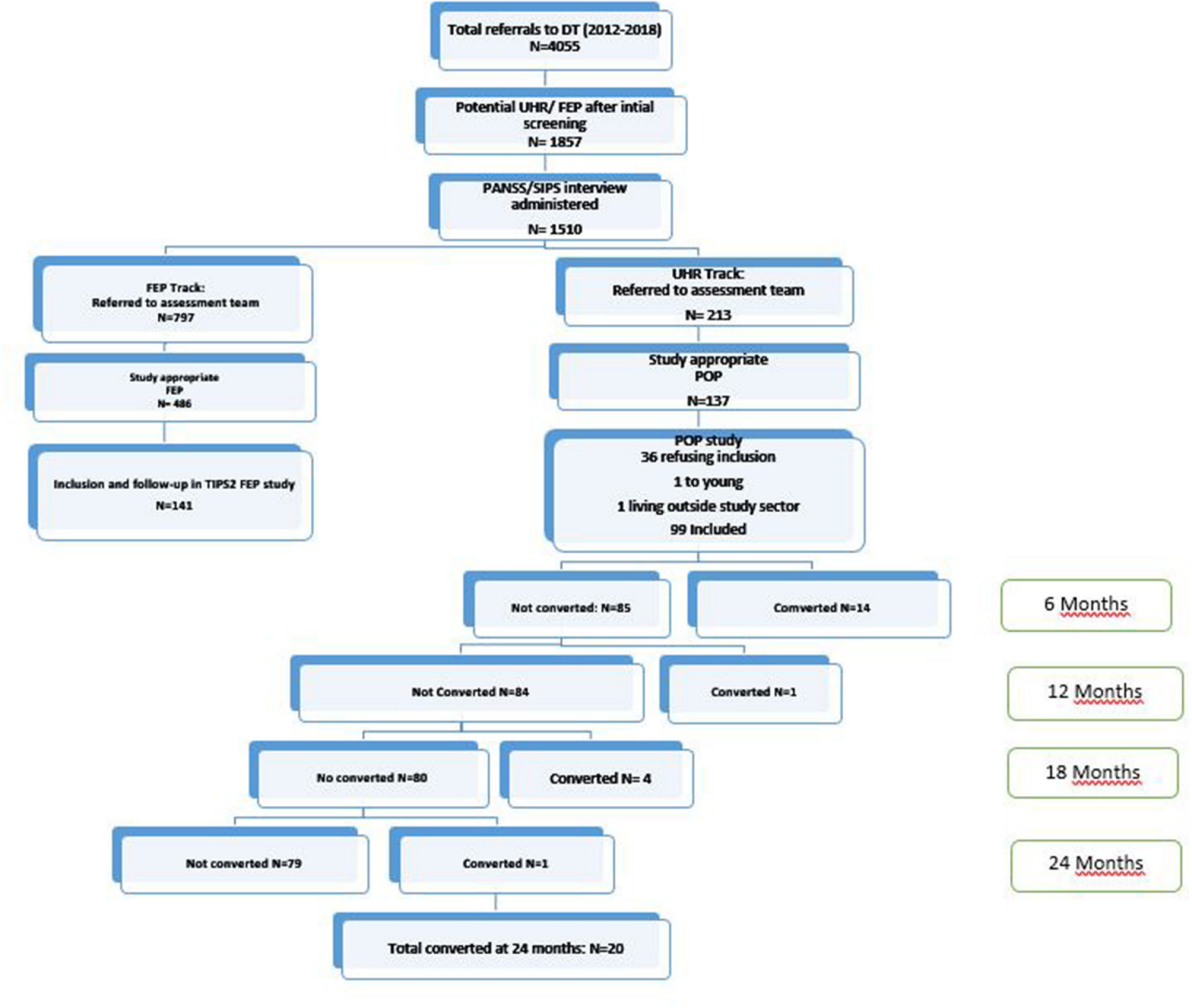
Study flow chart and psychosis conversions over time. DT, Detection team, FEP, first episode psychosis, UHR, ultra high risk, POP, prevention of psychosis; TIPS, early intervention for psychosis study; PANSS, positive and negative syndrome scale; SIPS, structural interview for prodromal syndromes.

The study inclusion period was from March 2012 until December 2018. Potential help-seeking UHR individuals in the catchment area could be referred to the DT by health care providers, educators, social service agencies, parents, caregivers, or by self-referral. Potential study participants were screened by telephone and/or a face-to-face interview and those scoring above a selected threshold on the Prodromal Questionnaire—Brief (PQ-B) were then invited for a SIPS interview. The DT recruitment process is depicted in Figure 1.

### Assessment Measures

#### Prodromal Questionnaire (PQ-Brief)

The PQ- B is a 21-item self report scale with yes or no questions and was used to screen potential participants. Individuals scoring above a cut-off > 3 were offered further symptom assessment. The PQ- B has been established as a reliable and valid screening tool, with 89% sensitivity and 58% specificity (31).

#### Structured Interview for Prodromal Syndromes (SIPS)

The SIPS interview assesses positive (5 items), negative (6 items), disorganized (4 items), and general symptoms (4 items) (7). The range of the Scale of Psychosis-risk Symptoms (SOPS) is 0–6, where a score of 0 represents the absence of symptoms, and a score of 6 represents a severe and psychotic state. APS is the presence of at least one of the items on the positive symptom subscale at a moderate (=3), moderately severe (=4), or severe, but not psychotic level (=5). BLIPS is defined as at least one positive symptom at the intensity of 6 with a duration of at least several minutes a day for a frequency of at least once a month. Finally, criteria for GRD are met when there is a genetic (familial) risk for any psychosis in the schizophrenia or affective spectrum plus a deterioration of daily life functioning. Known family history of psychiatric disorders in first degree relatives was registered based on clinical interview with participant at baseline. All SIPS interviews were conducted by one single expert rater, and interviews were discussed on weekly team meetings in order to reach consensus. Psychiatric nurses trained in using the SIPS conducted the SIPS interviews. Consensus regarding the UHR state was reached during weekly diagnostic meetings. Conversion to psychosis was defined as scoring 6 or above on one or more of the items P1–P5 (SOPS) indicating a “Severe and Psychotic” level of intensity of symptoms. For one participant, due to missing SIPS data, conversion was ascertained thorough patient medical file review.

#### Structured Clinical Interview for the Diagnostic and Statistical Manual-IV. Axis I Disorders (SCID)

The SCID interview (32) was used for diagnostic assessment and conducted by trained clinical researchers. Reliability of SCID in the research group was satisfactory at kappa = 0.9 in 2012 (33), and since then, regular reliability trainings were undertaken to avoid drift.

#### Alcohol/Drug Use Disorders Identification Tests (AUDIT/DUDIT)

Current alcohol abuse was assessed by the Alcohol Use Disorders Identification Test (AUDIT) (34). The AUDIT is a 10 item screening instrument, and cut-off score for alcohol misuse was defined as alcohol use more than three times per week (during the last 12 months). Current drug abuse was assessed using the Drug Use Disorders Identification Test (DUDIT). The DUDIT is an 11 item screening instrument, with reported satisfactory measures of reliability and validity (35–37). The cut-off score for substance abuse was any use of illegal substances (during the last 12 months).

#### Calgary Depression Scale for Schizophrenia

Depressive symptoms were assessed by the Calgary Depression Scale for Schizophrenia (CDSS) (38). The CDSS is a nine item scale (not present = 0, severe = 3) and the interview and rating scale are found to have good to high interrater reliability and validity (39, 40). A score of six or higher indicates probable depression (40).

#### Global Assessment of Functioning, Split Version

The Global Assessment of Functioning (GAF) split version (41) was administered at every session.

#### Assessement Time Points

The SIPS was administered to participants 13 times over 2 years, starting with baseline, then monthly for 6 months and then every 3 months until 2 years post baseline. The participants were diagnostically evaluated by SCID at baseline, 12 and 24 month along with the other outcome variable interviews.

#### Follow-Up Clinical Data

Participants who converted to a psychotic disorder during the study were excluded from the POP study from the time of conversion and were offered need-adapted treatment for first episode psychosis and also offered inclusion in the ongoing FEP study, TIPS 2 (2). We had consent to obtain clinical data on these participants at the 2 year follow-up from patient files, regardless of their consent to participate in TIPS.

### UHR Interventions

All study eligible individuals were offered clinical treatment in the specialized mental health inpatient and outpatient care of Stavanger University hospital, depending on their age and area of residence. They were offered individually adapted treatment containing the following elements: (1) One-to-one monitoring of clinical status, symptom levels (UHR and psychotic), risk profiles (suicidality, dangerousness), instrumental and social functioning; (2) One-to-one case management for clinical, familial, social and vocational crises, needs and deficits, continuing for as long as needed; (3) Individual cognitive behavioral therapy (CBT) (max of 26 sessions, once a week) addressing cognitive distortions and social impairments, and to maintain real world investment (based on the EDIE II study) (42); (4) Individuals were offered single family psycho-education treatment (6–12 months duration) to inform patients and families about current problems, how to understand and cope with them, especially within the family (43, 44); (5) Anti-anxiety agents and anti-depressants were available if indicated; and (6) Antipsychotic medication could be offered if the participant either entered the study with any SIPS positive symptom score of 5, or if any positive SIPS symptom score(s) moved from a 3 or 4 to a 5.

The study had ethical approval from the National Committee for Medical and Health Research Ethics (2009/949). Participation in the study was based on written informed consent. Parents or legal guardians gave informed consent for patients younger than 16 years of age.

### Statistics

The statistical analyses were performed in IBM SPSS Statistics v. 26 (45). Sample characteristics were obtained using simple descriptive statistics. Bivariate comparisons between those who converted to psychosis and those who did not were conducted employing student *T*-tests for independent samples; distributions of symptom scores were within the range of “approaching normality” for positive, negative, and general symptoms (skewness statistic 0.16; −0.05; and −0.45, respectively) and moderately skewed for disorganized symptoms (0.85). For categorical variables with more than one level (i.e., diagnostic categories), dummy variables were constructed in order to dichotomise them. For comparing converters to non-converters, Relative Risk was computed and due to small and uneven sample sizes, Fisher exact tests were chosen. Linear mixed model regression was used to estimate longitudinal conversion predictors. The dependent variable was conversion yes/no, and predictors (random factors) were SIPS symptom levels the first 6 months after inclusion in the study. Fixed factors were assessment time, age, and gender. A 6 months' time span was chosen as 70% of the patients who converted did so within the first 6 months. The model included age and gender and their interaction. For this analysis, we had access to information on conversion for 97 participants, and the following symptom data sets (N's reported): Baseline 96; 1 month 55; 2 months 55; 3 months 59; 6 months 55. Further, in order to estimate the association between time to conversion to psychosis, and symptom levels at identification and study inclusion, a cox regression model of proportional hazard was fitted. Age, gender, SIPS symptom levels (mean item scores on positive, negative, disorganized, and general symptoms), CDSS symptom levels (total score), and GAF level of symptoms and functioning were entered as independent (predictor) variables. Covariates were first entered stepwise, conditional, and then simultaneously (“enter”). Conversion yes/no was coded as the event for which the hazard ratio associated with covariates was estimated. Time to conversion in weeks was entered as the censored time variable. Regarding missing data for this analysis: Two participants lacked conversion data, and five lacked baseline symptom data. Finally, a longitudinal general linear mixed model (GLMM) of symptom development over time across conversion/non-conversion was fitted. As participants could re-enter the study after missing one or more assessments, missing assessments were counted from 1 month to 2 years as part of an attrition analysis conducted on the whole sample. The analysis compared participants with <6 missing assessments (over the course of the 2 years) to participants with more than six missing assessments. No significant differences were identified on baseline age, gender, or any of the four SIPS scales (sum-scores). Attrition thus appears to be random.

## Results

### Sample Characteristics

In the study period (2012–2018) 4,055 referrals were made to the DT. After initial screening the DT assessed 1,510 individuals either by PANSS or SIPS and assigned to the FEP TIPS2 study or the POP UHR study based on eligibility, (see Figure 1 for details). In the POP study we identified 141 UHR help-seeking individuals indicating an incidence of seven per 100,000 people per year. The baseline assessment was completed by 99 of these individuals. There were no age or gender differences between consenters and non-consenters. The mean age of the sample was 16.9 years (SD = 3.7) and 53% were female and 94% were of Nordic origin. Being a young sample they had attended school for a mean of 10.5 years (SD = 2.1). The majority (94%) was single and living with their family. Sixty-six per cent of the participants fulfilled DSM IV criteria for an affective or anxiety disorder at baseline. The level of alcohol use was low, with 53% reporting no lifetime use of alcohol and only one participant reporting alcohol misuse above cut-off. Fifteen per cent reported illegal drug use during the 12 months prior to baseline.

### Conversion

The 2 year psychosis conversion rate was 19.6 %; that is, 20 participants developed a psychotic disorder, of whom 14 (70%) converted within 6 months of baseline.

Thus, there was a clear tendency toward early conversion, with a median time to psychosis of 14 weeks. Table 1 outlines characteristics of participants who converted vs. non-converters. Those who converted had significantly higher levels of negative and general symptoms at baseline. There were no statistically significant differences between non-converters and converters in whether they participated in psychotherapy (84 vs. 65%), family groups (43 vs. 35%), or received medication (45 vs. 60%). Only four participants received second generation anti-psychotic medication in low doses, two converters and two non-converters. Other medication was primarily anti-depressants (32%) and anxiolytics (5%), with no significant differences on these medications between converters and non-converters. The majority of participants (64%) were treated in outpatient clinics only, and the mean length of hospital inpatient treatment over the 2 year period was 2.8 weeks (SD = 9.9). At 2 year follow-up, 50% of the converters were clinically remitted and non-psychotic according to information found in patient files.

**Table 1:** Characteristics across 2-year conversion/non-conversion.

	**Non-conversion (*****N*** **=** **79)**		**Conversion (*****N*** **=** **20)**		**Analysis**		
	**Mean**	***SD***	**Mean**	***SD***	***T***	***Df***	***P***
**Symptoms at baseline**							
Age	16.5	2.9	17.0	3.0	−0.69	94	0.49
SIPS positive	2.1	0.7	2.4	0.7	−0.8	91	0.42
SIPS negative	1.7	0.9	2.4	0.8	−3.1	89	**0.03**
SIPS disorganized	0.8	0.5	0.9	0.8	−0.5	88	0.62
SIPS general	2.2	0.9	2.6	0.09	−2.0	89	**0.05**
CDSS total score	7.6	4.9	9.6	4.8	−1.56	92	0.12
	*N*	%	*N*	%	RR	Exact sig. (2-sided)	
DSM IV diagnosis at baseline^*^							
Affective disorder	25	33	8	40	1.1	0.60	
Anxiety/OCD	27	35	3	15	0.9	0.11	
PTSD	3	4	3	15	1.6	0.10	
Developmental disorder	4	6	0	0	0.8	0.58	
Schizotypal disorder	1	1	0	0	0.8	1.00	
Substance use disorder	1	1	1	5	1.6	0.37	
Other	10	13	2	10	0.9	1.00	
Psychosis NOS	1	1	2	10	2.4	0.11	
Not fulfilling axis 1 criteria	5	7	1	5	1.3	1.00	
**Treatment over the 2-year study period**							
Psychotherapy	65	84	13	65	0.8	0.06	
Family groups	33	43	7	35	0.9	0.80	
Medication^*a*^	35	45	12	60	1.1	0.32	
**Gender**							
Female	40	52	12	63	1.1	0.45	
**Alcohol and substance misuse at baseline**							
Alcohol	1	1	0	0	0.8	1.00	
Illegal substance	12	16	2	11	0.9	1.00	

* *Missing data N = 3. Significant effects are marked in bold. ^a^Anti-psychotics: 2 persons who converted and 2 who did not; other medication concerned anti-depressants, anxiolytics or anti-ADHD medication*.

### Predictors of Conversion

**Figure 2:**
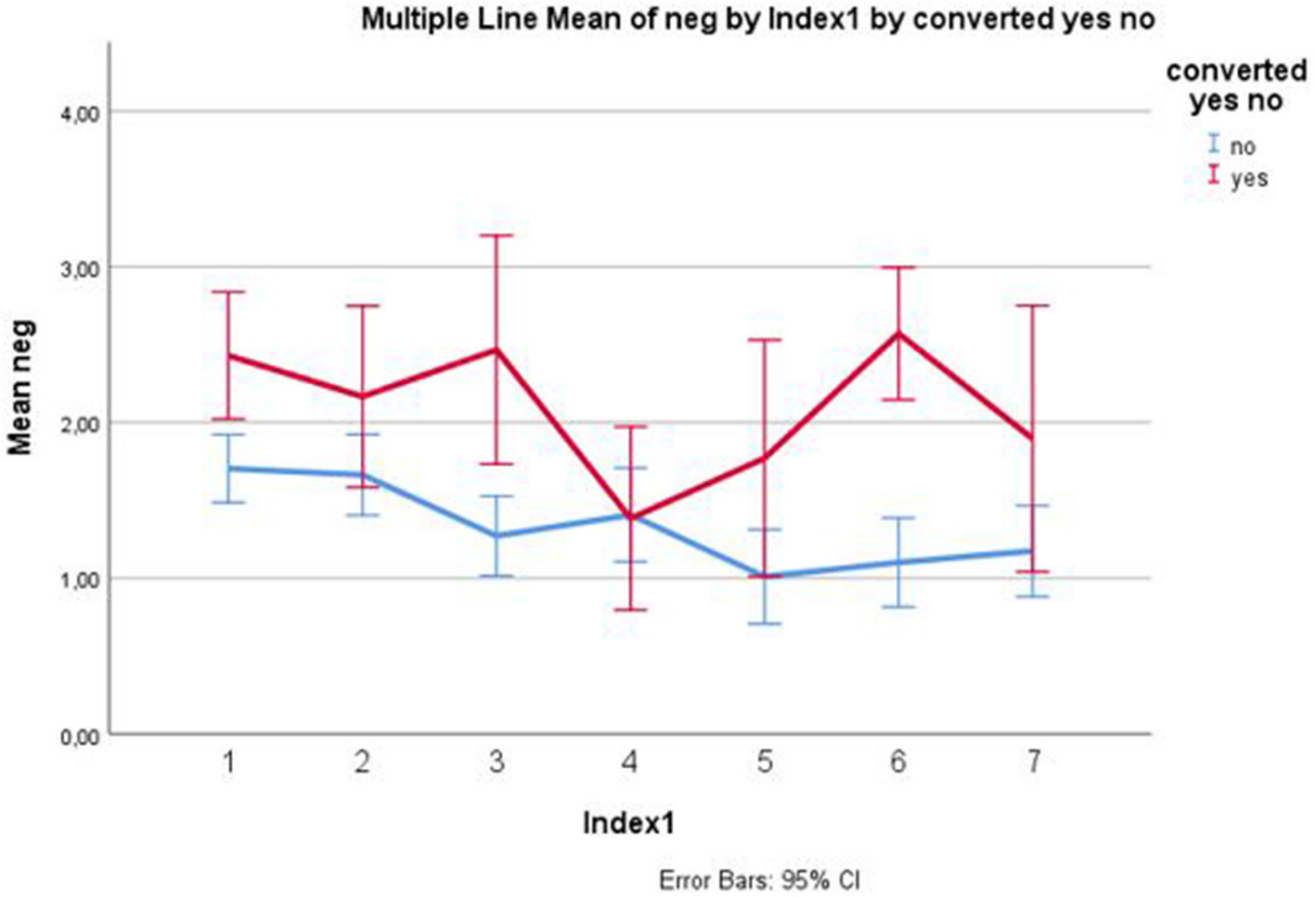
Negative symptoms 0–6 months converters/non-converters. Mean item scores and 95% confidence intervals. *X*-axis: 1 = baseline, 2 = 1 month, 3 = 2 month, 4 = 3 month, 5 = 4 month, 6 = 6 month, 7 = 6 month.

**Figure 3:**
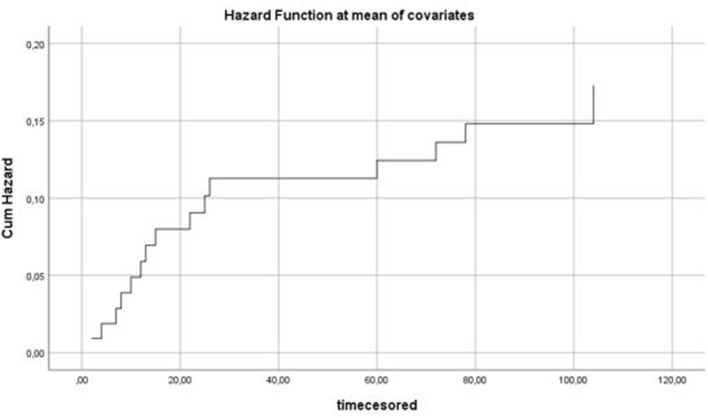
Hazard function of the association between predictors (age, gender, SIPS symptom levels, CDSS symptom levels, GAF symptom and GAF function) and time to conversion in weeks. *X*-axis: time in weeks. *Y*-axis: Cumulative hazard.

In the longitudinal analyses, employing symptom data over time in order to predict conversion, the course of positive and negative symptoms significantly predicted conversion; no other symptom courses were significant (Table 2). The development of negative symptoms is illustrated in Figure 2. A higher age also significantly contributed, and there was an interaction effect with gender: a higher age in females contributed more to the model than a higher age in males. In the stepwise cox regression model, only baseline negative symptom levels were associated with time to conversion (hazard ratio 2.2; 95% confidence interval 1.2 – 3.9; significance level of model *p* < 0.08). The hazard function is depicted in Figure 3.

**Table 2:** Longitudinal predictors of conversion to psychosis.

**Variable**	**Type II sum of squares**	***df***	**Mean square**	***F***	**Sig**
Corrected model	19.18	24	0.799	7.40	0.000
Intercept	0.006	1	0.006	0.055	0.816
SIPS positive	0.468	1	0.048	4.33	**0.038**
SIPS negative	0.618	1	0.618	5.73	**0.017**
SIPS disorganized	0.006	1	0.06	0.058	0.810
SIPS general	0.03	1	0.003	0.030	0.862
GAF	0.000	1	0.000	0.004	0.948
Age	11.317	11	1.029	9.526	**0.000**
Gender	0.014	1	0.014	0.128	0.720
Age*gender	5.621	7	0.752	6.659	**0.000**

Significant effects are marked in bold. Age*gender interaction term.

## Discussion

The main finding of this study was that we were able to identify 141 UHR help-seeking individuals, indicating a yearly incidence of seven per 100,000 people, compared to our prediction of 90 per 100,000 people. It is important to note that despite the implementation of information campaigns for detection, our study included help seeking individuals rather than being restricted to a broad population level campaign. Thus we detected around 8% of the expected cases compared with 4–5% of cases reported in a recent meta review (1). It is expected that the incidence of UHR would be higher in the risk enriched environment of mental health care compared to the general community. Even though one of the targeted information strategies was aimed at providing education for clinicians in the specialized mental health services on signs and symptoms of the UHR phase, the study was not able to reach the expected UHR incidence level. Study findings reflect previous UHR research, showing a low referral rate of potential UHR individuals to UHR detection centers, since many individuals are already being treated in other secondary mental health care services (20). There is a need for improved tools for case detection such as the proposed individualized transdiagnostic risk calculator (21, 22).

Where reducing DUP is achieved through targeted information about specific symptoms such as hallucinations or delusions, this is currently not possible in UHR due to the non-specificity of early emerging symptoms. However, the treatment provided in our study was not aimed solely at preventing conversion to psychosis but also at improving psychosocial functioning and family relations through CBT, psychotherapy and family work. Medication (anti-anxiety, antidepressants and antipsychotic medication) was available according to the treatment algorithm if needed and the clinicians recommended such use with 47% of the sample reported using such medication during the 2 year follow up.

Our sample was young, mostly adolescents, with a mean age of 16.9 years whereas a comprehensive review of 42 studies showed a mean age of UHR/CHR participants of 20.6 years (1). While we had equal representation of males and females, the review showed a predominance of males. Depression and anxiety were the most common mental health problems for our sample as indicated by diagnostic category and the Calgary Depression Scale for Schizophrenia (CDSS) scores (40), averaging above cutoff at baseline.

The 2 year conversion rate of 20% is consistent with other studies (1, 46). It should be noted that individuals who developed a FEP were immediately offered treatment for psychotic symptoms and thus had a DUP of 0 weeks. The SIPS negative symptoms subscale emerged as the symptom dimension significantly associated with conversion to psychosis in all analyses. This warrants some discussion, as conversion is defined by positive, not negative, symptoms. However, social withdrawal is a central feature in negative symptoms. In youth, healthy psychosocial development commonly occurs in social arenas, such as school or college, sports clubs, friend groups, and romantic involvements (47). Emerging psychological problems of a severe nature hamper the social functioning essential for relationships with peers (48). Hence, we argue that when mental health problems reach a level at which the interaction with peers becomes compromised, this may be indicative of more severe psychological distress, and in some cases, psychosis.

Finally we are not able to state that the POP early detection program resulted in an increase in the number of detected and successfully treated UHR individuals to a level needed to influence the incidence of FEP, as the treated incidence of FEP was stable in catchment area across the time-period. This will be a focus for further studies.

## Strengths and Limitations

Our study capitalized on the strengths of the TIPS longitudinal cohort study to implement one of the first large scale information campaigns targeting young people at UHR for psychosis. Contrary to many other UHR studies, we targeted both community and clinical populations. Our study provides important learning for all UHR researchers regarding the process of recruitment and retention of this young and vulnerable population. The primary limitations of our study concern generalisability and representativeness. A small sample size and a relatively high attrition rate limit generalisability. Attrition does however appear to be at random, and the sample can be assumed to be representative with regard to baseline characteristics.

## Conclusions

In conclusion, the early detection strategies employed were not able to detect UHR cases to the extent predicted by the population statistics and further case enrichment appears to be needed to improve this. Enhanced screening focusing on subclinical psychotic experiences and social anhedonia in the adolescent general population might bring more UHR individuals into clinical services (49, 50). As negative symptoms were significant predictors of transition to psychosis, a greater focus on negative or negative-like symptoms and social withdrawal seems warranted. The use of social media may to an extent mask social withdrawal, and we thus propose to expand the use of these platforms in UHR detection work. Further, we propose combining this with the use of enhanced screening tools like the individualized transdiagnostic risk calculator, possibly with an addition of social withdrawal (21, 22). Finally, we propose that social media and other awareness campaigns be focused on the general population, whereas enhanced tools for detection be used in risk enriched environments, predominantly specialized adolescent mental health services. This could improve UHR detection rates and enhance help-seeking behavior in the target population.

## Data Availability Statement

The datasets presented in this article are not readily available because according to Norwegian law, data sharing requires approvals from the Regional Committees for Medical and Health Research Ethics, and from the Data Protection Officer at Stavanger University Hospital, on the basis of specific research proposals. Requests to access the datasets should be directed to inge.joa@sus.no.

## Ethics Statement

The studies involving human participants were reviewed and approved by National Committee for Medical and Health Research Ethics (2009/949). Participation in the study was based on written informed consent. Parents or legal guardians gave informed consent for patients younger than 16 years of age.

## Author Contributions

IJ, WH, and JJ contributed to conception and design of the study as well as data collection. JL and MW also contributed to the data collection. WH conducted the statistical analysis. IJ wrote the first draft of the manuscript. All authors contributed to manuscript revision, read, and approved the submitted version.

## Conflict of Interest

The authors declare that the research was conducted in the absence of any commercial or financial relationships that could be construed as a potential conflict of interest.
